# Preparation process and properties of polyurethane/phosphogypsum-modified asphalt and its mixtures

**DOI:** 10.1371/journal.pone.0327312

**Published:** 2025-07-11

**Authors:** Jinshun Xue, Yu Yang, Junfeng Li, Wei Zhao, Yuanyuan Wang

**Affiliations:** 1 Hubei Key Laboratory of Vehicle-infrastructure Cooperation and Traffic Control, Xiangyang, China.; Hubei Key Laboratory of Power System Design and Test for Electrical Vehicle, Hubei University of Arts and Science, Xiangyang, China.; School of Civil Engineering and Architecture, Hubei University of Arts and Science, Xiangyang, China; 2 Henan Xingmi Expressway Co., Ltd, Zhengzhou, China; 3 HPCPDI Ruizhi Transportation Technology Consulting Co., Ltd., Hebei Province Road Structure and Material Technology Innovation Center Shijiazhuang, Shijiazhuang, China; 4 School of Civil Engineering and Architecture, Hubei University of Arts and Science, Xiangyang, China; Shandong University of Technology, CHINA

## Abstract

The preparation process parameters and composition of polyurethane/phosphogypsum-modified asphalt were studied to achieve comprehensive utilization of phosphogypsum and reduce asphalt pavement costs while ensuring performance compliance. Additionally, the aging resistances of the modified asphalt and its mixtures were investigated. The results showed that the order of the influence of the different preparation process parameters on the performance of modified asphalt is preparation temperature, storage time, reaction time, and shear rate. The optimal preparation process parameters, namely 130°C preparation temperature, 2000 rpm shear rate, 1.5 h reaction time, and 1.0 h storage time, of polyurethane/phosphogypsum composite-modified asphalt are recommended based on the experimental results. Polyurethane/phosphogypsum-modified asphalt with 3% component A, 1% component B, 5% phosphogypsum, and 0.5% coupling agent exhibited superior performance. The average change rates of the modified asphalt properties after short- and long-term aging were 23.9% and 42.2% lower than those of the base asphalt, respectively. Finally, the modified asphalt mixture exhibited average change rates of 8.7% and 18.2% in high-temperature performance after short- and long-term aging, respectively, which were significantly lower than the change rates of 15.3% and 23.5% observed in the base asphalt mixture.

## Introduction

Progress in road engineering has imposed increased demands on pavement performance and materials [1]. Conventional asphalt materials are inadequate for high-grade pavements, necessitating asphalt modifications to enhance road performance [2]. Common polymer-based asphalt modifiers include SBS, crumb rubber, and SBR [3–5]. Polyurethane, which is a novel polymer, exhibits superior wear resistance, low-temperature tolerance, aging resistance, and structural advantages [6]. Recent studies have explored its potential as a substitute for traditional polymer modifiers in asphalt modification. Sun et al. investigated the preparation process and rheological properties of polyurethane-modified asphalt. Their findings indicated that chemical reactions occur during preparation, enabling polyurethane to dissolve and disperse asphaltenes within the base asphalt and forming a more stable system. This enhances the high-temperature performance while reducing the sensitivity to temperature fluctuations and loading frequency [7–8]. Liufu et al. studied the mix design and pavement performance of polyurethane-modified asphalt mixtures. The results demonstrated that polyurethane-modified mixtures exhibit significantly superior high-temperature stability and low-temperature crack resistance compared with base asphalt and SBS-modified asphalt, with water stability comparable to that of SBS-modified mixtures [9–10].

Phosphogypsum, which is a byproduct of wet-process phosphoric acid production generated via sulfuric acid decomposition of phosphate rock, contains harmful impurities and incurs high purification costs [11]. Its massive stockpiling poses severe environmental risks, driving the urgency for effective utilization [12–13]. Current applications focus on its use in cementitious materials or as an additive in cement mortar [14–16]. Several studies have revealed that increasing the phosphogypsum content enhances the strength and softening coefficient of phosphogypsum-based composite cementitious materials; however improvements in its water resistance properties remain limited. The co-incorporation of quicklime and silica fume significantly improves the compressive strength and water resistance of high-temperature-modified phosphogypsum-slag-cement mortar [17]. Phosphogypsum is primarily used as a subgrade additive in road engineering [18]. Ji et al. developed a phosphogypsum stabilizer using sodium methyl silicate, sodium silicate, and emulsifiers to enhance the strength, water stability, and environmental safety of subgrade fillers, thereby meeting the technical requirements for highway embankments [19]. Shi et al. proposed a feasible mix ratio (red mud:cement:lime = 100:8:2) for cement-lime-phosphogypsum-stabilized red mud as an alternative to traditional base materials [20]. Granulation technology was adopted to achieve phosphogypsum-based cold-bonded aggregates. The results of experiments on the physical properties, mechanical strength, impurity stabilization ability, and microstructure showed that the aggregates with a phosphogypsum content up to 80% were qualified lightweight aggregates [21]. Additionally, the feasibility of using phosphogypsum as an alternative mineral powder in asphalt mixtures has been investigated. An asphalt mixture with phosphogypsum can improve the high-temperature properties and reduce the fatigue life of asphalt mixture [22]. The calcium and sulfur in phosphogypsum may act as fillers or participate in chemical interactions within the asphalt, improving the high-temperature performance by elevating the softening point and reducing deformation [23–24]. However, the aging resistance of polyurethane/phosphogypsum composite-modified asphalt and its mixtures remains unexplored. This study proposes a composite modification approach using polyurethane (providing elasticity and flexibility) and phosphogypsum (enhancing the rigidity and high-temperature stability). As an inorganic material, phosphogypsum may also improve the compatibility between asphalt and mineral aggregates, thereby bolstering the overall stability of the mixtures [25]. Although polyurethane may increase material costs, its composite use with phosphogypsum, which is an industrial byproduct, balances the overall expenses and enhances the economic viability.

This study aims to optimize the composition and preparation process parameters of polyurethane/phosphogypsum composite-modified asphalt, followed by an evaluation of its aging resistance and that of its mixtures. The goal is to achieve comprehensive utilization of solid waste resources and reduce asphalt pavement material costs while ensuring performance compliance.

## 1. Materials and test program

### 1.1. Materials

(1) Asphalt

The asphalt binder used in this study was 90# base asphalt; its technical indicators are presented in Table 1.

**Table 1:** Technical indicators of 90# base asphalt.

Test items	Test values	Required values
25°C Penetration (0.1 mm)	92.2	90 ~ 110
10°C Ductility (cm)	112	≥25
Softening point (°C)	46.5	≥42

(2) Aggregates

Limestone-crushed stones were used for the asphalt mixtures in this study; the technical indicators of the different aggregate sizes are listed in Table 2.

**Table 2:** Technical indicators of coarse aggregate.

Test items	Test values of different aggregates			Standard values
	9.5-16 mm	4.75 - 9.5 mm	2.36 - 4.75 mm	
Density (g/cm3)	2.672	2.699	2.700	≥2.6
Water absorption (%)	0.766	1.041	–	≤2.0

(3) Polyurethane materials

**Fig 1:**
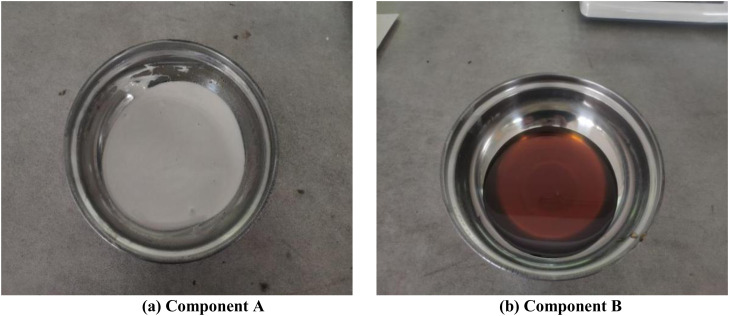
Polyurethane sample (a) Component A (b) Component B.

The polyurethane used in this study consisted of two components: component A as the main agent and component B as the curing agent (Fig 1). Component B, which contains isocyanate groups (-NCO), serves as the key reactive ingredient in polyurethane curing. It reacts with component A (polyols) and the active groups (e.g., hydroxyl and amino) in asphalt to form cross-linked urethane structures that directly influence the mechanical strength, viscoelasticity, and thermal stability of the modified asphalt. Both components are oily, with viscosities of 21000 and 100 mPa•s, respectively.

(4) Phosphogypsum

**Fig 2:**
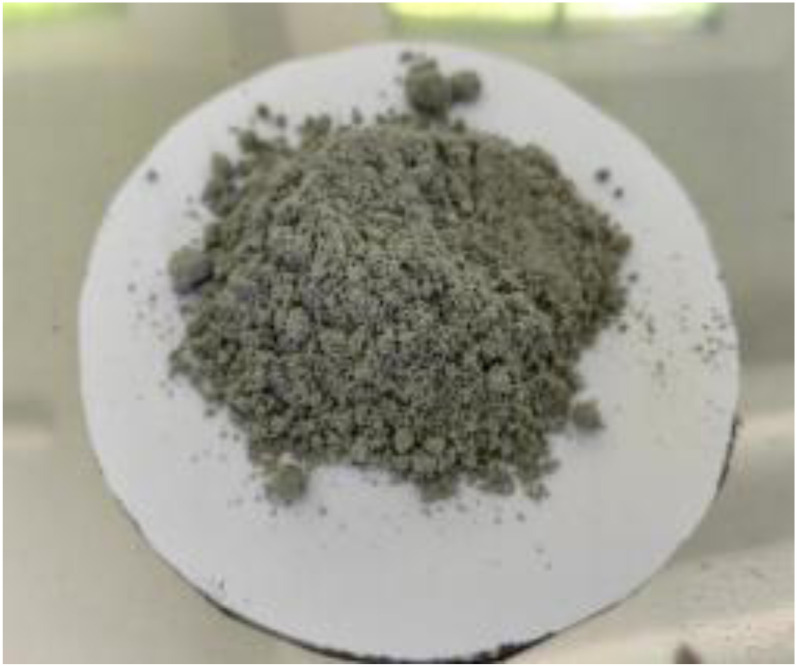
Phosphogypsum sample.

Phosphogypsum is a solid waste generated during the production of phosphoric acid via the treatment of phosphate ore with sulfuric acid. Its main component is calcium sulfate, which can enhance the durability of asphalt. A phosphogypsum sample is shown in Fig 2. To achieve a moisture-free condition, the phosphogypsum specimens were oven-dried at 50°C for 24 hours before conducting the experiments.

(5) Coupling agent

**Fig 3:**
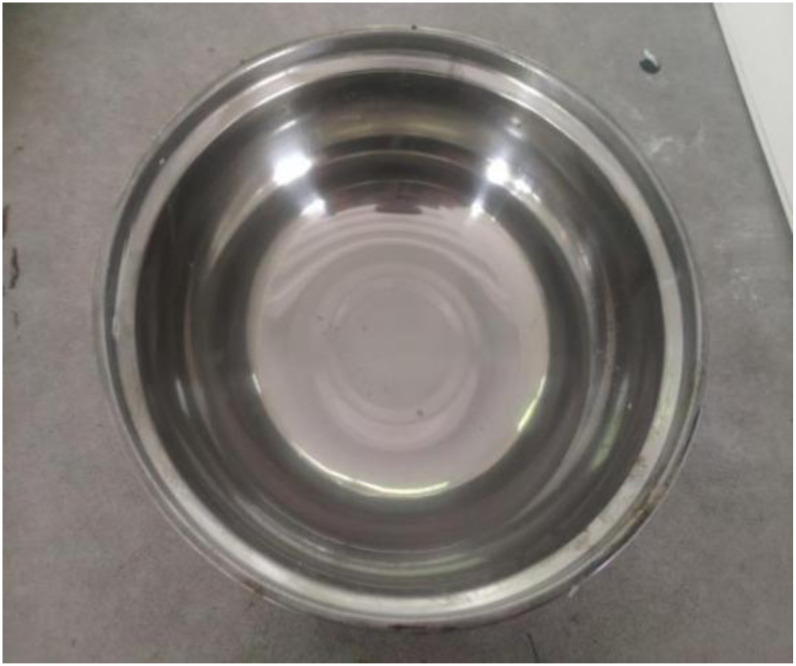
Coupling agent sample.

The coupling agent samples used in this study are shown in Fig 3. Coupling agents can enhance the interfacial compatibility between inorganic fillers (phosphogypsum) and the organic matrix (asphalt–polyurethane system) through physical adsorption or weak chemical bonding.

### 2.2. Preparation process of modified asphalt

The process of preparing the polyurethane/phosphogypsum-modified asphalt is as follows:

Step 1: The base asphalt is heated to a molten state at 120‒140°C and polyurethane material component A is added. The mixture is stirred for 5 min using a shear mixer, resulting in mixture A, while ensuring its temperature remains within 120‒140°C.

Step 2: Polyurethane material component B is added to mixture A, and the mixture is stirred for 5 min using a shear mixer, resulting in mixture B, while maintaining its temperature within 120‒140°C.

Step 3: Phosphogypsum is added to mixture B, and the mixture is stirred for 5 min using a shear mixer, resulting in mixture C, while maintaining its temperature within 120‒140°C.

Step 4: The coupling agent is added to mixture C, and the mixture is stirred for a predetermined preparation time using a shear mixer, resulting in polyurethane phosphogypsum composite-modified asphalt. The temperature is maintained within 120‒140°C for this step.

Step 5: The prepared polyurethane phosphogypsum composite-modified asphalt is cured in an 120‒140°C oven, resulting in polyurethane phosphogypsum composite-modified asphalt.

## 3. Optimal preparation process parameters of polyurethane/phosphogypsum composite-modified asphalt

The four preparation parameters – preparation temperature, shear rate, reaction time, and storage time – have been identified as critical factors significantly influencing the performance characteristics of modified asphalt [26]. Four preparation process parameters, namely the preparation temperature, shear rate, reaction time, and storage time, were evaluated in this study to investigate the effects of the preparation process on the performance of the polyurethane/phosphogypsum composite-modified asphalt. The optimal preparation process parameters for the composite-modified asphalt are recommended based on the experimental results.

An orthogonal experimental design was adopted to ensure the reliability of the experimental results while achieving a balance between experimental efficiency and comprehensiveness. The different preparation process parameter levels are listed in Table 3, and the experimental schemes are listed in Table 4.

**Table 3:** Preparation process parameter levels of orthogonal experimental design.

Levels	Preparation temperature (°C)	Shear rate (rmp)	Reaction time (h)	Storage time (h)
I	120	1500	0.5	0.5
II	130	2000	1.0	1.0
III	140	2500	1.5	1.5
IV	150	3000	2.0	2.0

**Table 4:** The experimental schemes of different preparation process parameters.

No.	Preparation temperature (°C)	Shear rate (rmp)	^Reaction time (h)^	Storage time ^(h)^
1	120	1500	0.5	0.5
2	120	2000	1.0	1.0
3	120	2500	1.5	1.5
4	120	3000	2.0	2.0
5	130	2000	0.5	1.0
6	130	2500	1.0	0.5
7	130	1500	1.5	2.0
8	130	3000	2	1.5
9	140	3000	0.5	1.5
10	140	2500	1.0	2.0
11	140	2000	1.5	0.5
12	140	1500	2.0	1.0
13	150	2000	0.5	2.0
14	150	1500	1.0	1.5
15	150	3000	1.5	1.0
16	150	2500	2.0	0.5

**Fig 4:**
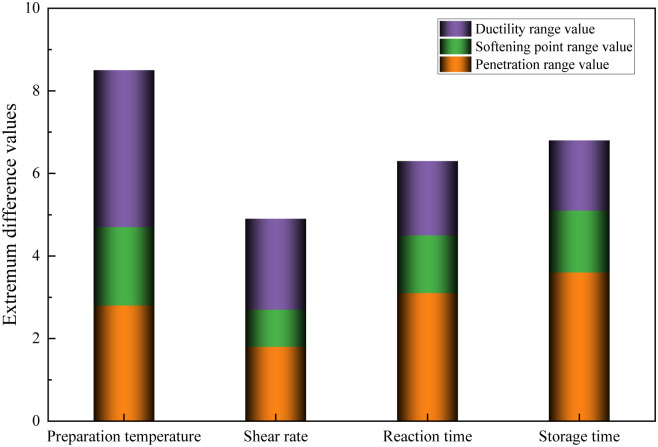
Extremum difference normalized values of different preparation parameters test results.

The extremum difference values of the preparation parameter test results were normalized to analyze the influence of the preparation processes on the performance of the modified asphalt. The extremum difference normalized value results are shown in Fig 4.

Fig 4 shows that differences existed in the extremum difference normalized values of the penetration, softening point, and ductility under different preparation process parameters. These extremum difference normalized values were summarized to evaluate the influence of the parameters on the performance of the modified asphalt comprehensively. The results showed that these values were the largest, followed by the storage time and reaction time. The extremum difference normalized values of the different shear rates were the smallest. The larger the extremum difference normalized values under different preparation process parameters, the higher their impact on the performance of the modified asphalt. This indicates that the order of influence of the different preparation process parameters on the performance of the modified asphalt is preparation temperature, storage time, reaction time, and shear rate. Thus, the influence of the preparation temperature, storage time, and reaction time on the performance of the modified asphalt should receive more attention when optimizing the preparation process parameters. The preparation temperature exerted a dominant influence on the compatibility between the base asphalt, polyurethane, and phosphogypsum, critically governing the formation of a three-dimensional network structure within the modified asphalt. A temperature deviation of 5°C could induce fluctuations of 3–5°C in softening point and reductions of 10–15% in ductility‌ [26]. Extended storage and reaction times moderately enhanced the interfacial compatibility between the base asphalt, polyurethane, and phosphogypsum; however, their effects in the molecular activation mechanism were inferior to those of the preparation temperature. Additionally, shear rate was primarily observed during the physical blending phase, contributing minimally to the chemical modification processes, and thus exhibiting the least impact on the performance of the modified asphalt.

### 3.1. Effect of preparation temperature on performance of modified asphalt

**Fig 5:**
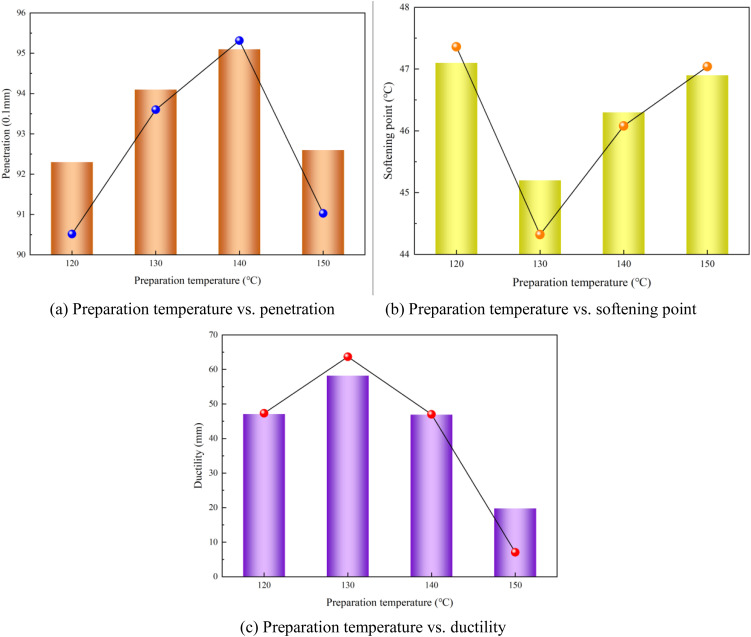
Preparation temperature vs. modified asphalt performances (a) Preparation temperature vs. penetration (b) Preparation temperature vs. softening point (c) Preparation temperature vs. ductility.

Fig 5 shows the correlation between the preparation temperature and performance parameters of the polyurethane/phosphogypsum-modified asphalt (including penetration, softening point, and ductility). The penetration initially increased and then decreased with an increasing preparation temperature, and the softening point exhibited an inverse trend, reaching its minimum value (45.2°C) at 130°C. Notably, peak ductility (58.2 cm) was observed at this critical temperature. A comprehensive analysis indicated that the optimal preparation temperature range was between 120°C and 130°C, where the modified asphalt achieved balanced performance metrics. The penetration maintained values within the 92–94 (0.1 mm) range, ensuring appropriate viscosity for construction workability; the softening point stabilized above 45°C, meeting high-temperature stability requirements; and the ductility preserved the flexibility, with values exceeding 47 cm, thereby guaranteeing low-temperature crack resistance. This temperature window aligns with the thermodynamic requirements for polymer dispersion optimization and minimizes the thermal degradation risks of the polyurethane modifiers. The observed performance inflection at 130°C corresponds to the equilibrium point between molecular activation energy and thermal oxidative aging kinetics.

### 3.2. Effect of storage time on performance of modified asphalt

**Fig 6:**
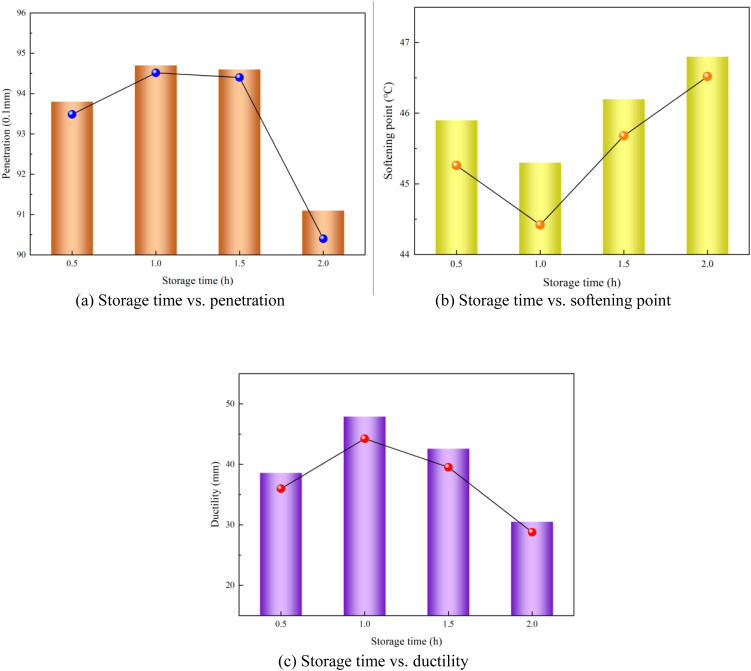
Storage time vs. modified asphalt performances (a) Storage time vs. penetration (b) Storage time vs. softening point (c) Storage time vs. ductility.

The relationship between the storage time and modified asphalt performance is shown in Fig 6. Both penetration and ductility initially increased and then decreased with an increasing storage time, peaking at a storage time of 1.0 h (penetration: 9.47 mm; ductility: 47.9 cm). This trend aligns with the time-dependent molecular rearrangement of the modified asphalt during early-stage storage. The softening point demonstrated inverse parabolic behavior, reaching the minimum value (45.3°C) at the 1.0 h mark before gradually increasing with prolonged storage time. This temporal optimization mechanism reflects the competition between polymer network maturation (beneficial) and thermal degradation kinetics (detrimental).Experiments confirmed that an optimal balance between the rheological functionality (penetration/ductility maxima) and durability (controlled softening point elevation) was achieved after 1.0 h. Overall, the prepared modified asphalt exhibited improved performance when the storage time was 1.0 h.

### 3.3. Effect of reaction time on performance of modified asphalt

**Fig 7:**
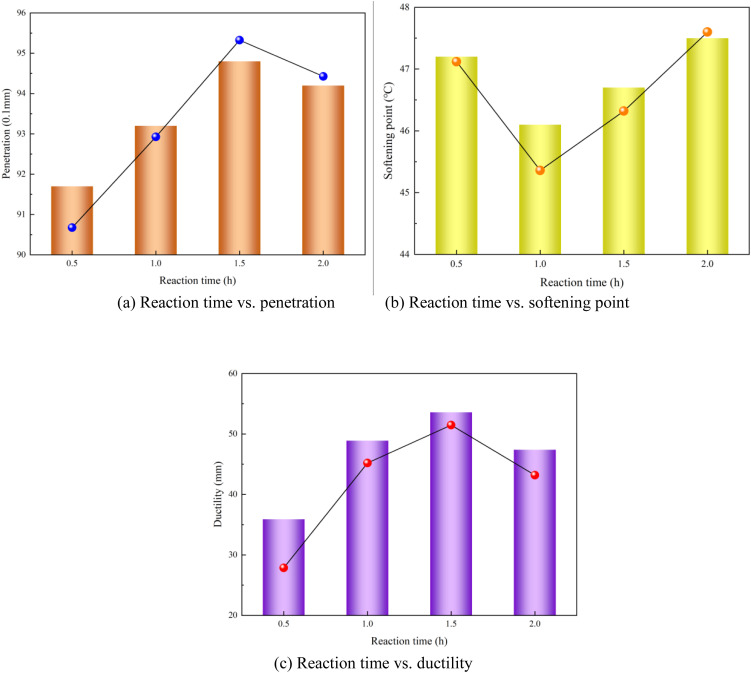
Reaction time vs. modified asphalt performances (a) Reaction time vs. penetration (b) Reaction time vs. softening point. **(c)** Reaction time vs. ductility.

The performance evolution of polyurethane/phosphogypsum-modified asphalt with the reaction time is illustrated in Fig 7. The rheological properties of the modified asphalt exhibited time-dependent nonlinear behavior during preparation, and penetration and ductility demonstrated an initial increase followed by a decrease, with the penetration peaking at ‌a reaction time of 1.5 h. The softening point exhibited an inverse parabolic trend, reaching the minimum value at 1.5 h before gradually increasing with a prolonged reaction duration. At a reaction time of 1.5 h, the penetration (9.48 mm) ensured optimal viscosity for construction compatibility, the ductility (53.6 cm) maintained flexibility under low-temperature conditions, and the softening point stabilized above 46.2°C, meeting the thermal stability requirements. This temporal threshold balanced the polymer-modifier activation (enhancing the penetration/ductility) and thermal degradation initiation (affecting the softening point). Experiments confirmed 1.5 h as the critical juncture for achieving synergistic performance metrics in polyurethane/phosphogypsum-modified asphalt systems.

### 3.4. Effect of shear rate on performance of modified asphalt

**Fig 8:**
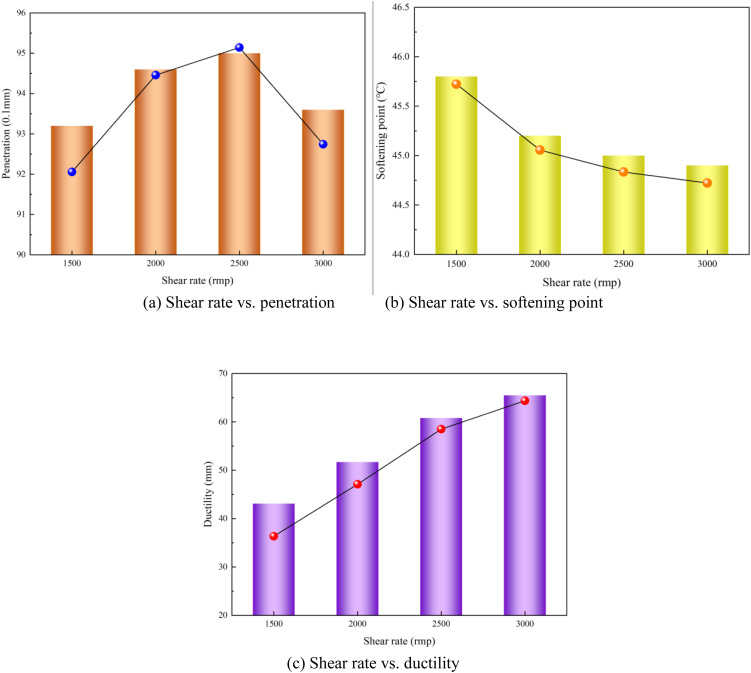
Shear rate vs. modified asphalt performances (a) Shear rate vs. penetration (b) Shear rate vs. softening point (c) Shear rate vs. ductility.

The shear rate-dependent performance of the modified asphalt is depicted in Fig 8. The rheological properties of the modified asphalt also exhibited significant shear rate dependence. The penetration initially increased, peaked at a shear rate of 2500 rpm, and then declined owing to polymer-chain disentanglement under an excessive shear rate. The softening point decreased monotonically, aligning with pseudoplastic fluid behavior, where the viscosity inversely correlated with the shear rate. The ductility improved progressively, which is attributed to enhanced modifier dispersion and reduced agglomeration at higher shear rates. Overall, when the shear rate was 2000 rmp, the prepared modified asphalt exhibited improved performance (penetration: 9.46 mm; softening point: 45.2°C; ductility: 58.2 cm). This critical shear threshold reflects the equilibrium between the modifier dispersion efficiency and polymer degradation initiation, optimizing both the mechanical and rheological performance in modified asphalt systems.

As mentioned previously, the optimal preparation process parameters (130°C preparation temperature, 2000 rpm shear rate, 1.5 h reaction time, and 1.0 h storage time) for polyurethane/phosphogypsum composite-modified asphalt are recommended.

## 4. Optimal composition of polyurethane/phosphogypsum composite-modified asphalt

Based on the optimal preparation process parameters of the modified asphalt, the influence of different compositions on the performance parameters (including penetration, softening point, and ductility) of the polyurethane/phosphogypsum composite-modified asphalt was investigated and the optimal composition is recommended.

The basic materials for the modified asphalt were 90# base asphalt, phosphogypsum, and polyurethane, which were composed of the main agent component A and curing agent component B. Coupling agents were used in this study to enhance the interfacial bonding between the base asphalt, phosphogypsum, and polyurethane. Coupling agents can improve material compatibility by bridging polarity differences, ensuring uniform dispersion of modifiers and increasing the storage stability by preventing segregation [27].

The different compositions of polyurethane/phosphogypsum composite-modified asphalt are listed in Table 5, and the experimental performance schemes of the modified asphalt are shown in Table 6.

**Table 5:** Composition levels of orthogonal experimental design.

Levels	Component A content (%)	Component B content B (%)	Phosphogypsum content (%)	Coupling agent content (%)
I	0	0	0	0
II	2	1	3	0.5
III	4	2	6	1.0
IV	6	3	9	1.5

**Table 6:** The experimental schemes of different compositions of modified asphalt.

No.	Component A content (%)	Component B content B (%)	Phosphogypsum content (%)	Coupling agent content (%)
1	0	0	0	0
2	0	1	3	0.5
3	0	2	6	1.0
4	0	3	9	1.5
5	2	2	0	1.5
6	2	3	3	0
7	2	0	6	1.5
8	2	1	9	1.0
9	4	3	0	1.0
10	4	2	3	1.5
11	4	1	6	0
12	4	0	9	0.5
13	6	1	0	1.5
14	6	0	3	1.0
15	6	3	6	0.5
16	6	2	9	0

**Fig 9:**
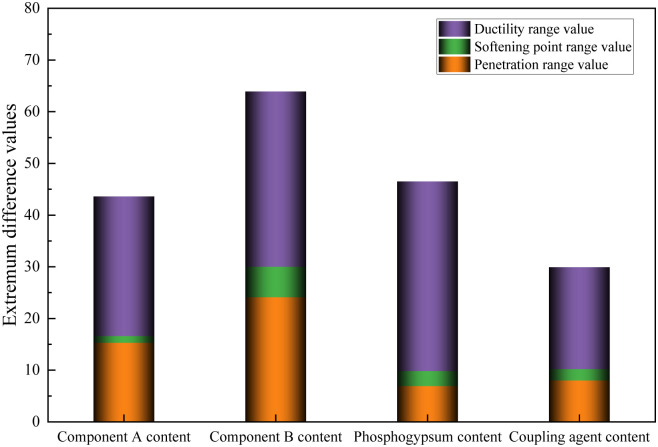
Extremum difference normalized values of different compositions test results.

The extremum difference values of the different composition test results were normalized to analyze the influence of different compositions on the performance of the modified asphalt. The extremum difference normalized value results are shown in Fig 9.

As illustrated in Fig 8, the extremum difference normalized values of the modified asphalt performance varied across different compositions. Notably, the values for the penetration and ductility exhibited significant differences, whereas those for the softening point showed relatively smaller variations. This indicates that changes in the composition of the polyurethane/phosphogypsum composite-modified asphalt exerted a greater influence on penetration and ductility. Furthermore, modified asphalt with varying component B (polyurethane curing agent) contents exhibited the highest extremum difference normalized values, followed by modified asphalt with different component A (polyurethane main agent) and phosphogypsum contents. Modified asphalt with varying coupling agent contents exhibited the smallest extremum difference normalized values. These results confirm that the order of influence of the composition on the modified asphalt performance is as follows: component B > component A > phosphogypsum > coupling agent. This hierarchy is attributed to the presence of component B, which serves as the key reactive ingredient in polyurethane curing. It reacts with component A (polyols) and active groups (e.g., hydroxyl and amino) in the asphalt to form cross-linked urethane structures that directly influence the mechanical strength, viscoelasticity, and thermal stability of the modified asphalt. Although component A participates in cross-linking reactions, its reactivity is typically lower than that of component B. The calcium sulfate in phosphogypsum may engage in acid–base reactions, modulate the pH of the asphalt system, and indirectly influence polyurethane curing rates. However, its impact is subordinate to the direct cross-linking effect of component B. Coupling agents primarily enhance the interfacial compatibility between the inorganic fillers (phosphogypsum) and organic matrix (asphalt–polyurethane system) via physical adsorption or weak chemical bonding. This interfacial optimization contributed minimally to the overall performance enhancement of the modified asphalt. This performance hierarchy aligns with the chemical reactivity and functional roles of the compositions within the composite system.

### 4.1. Effect of component A content on performance of modified asphalt

**Fig 10:**
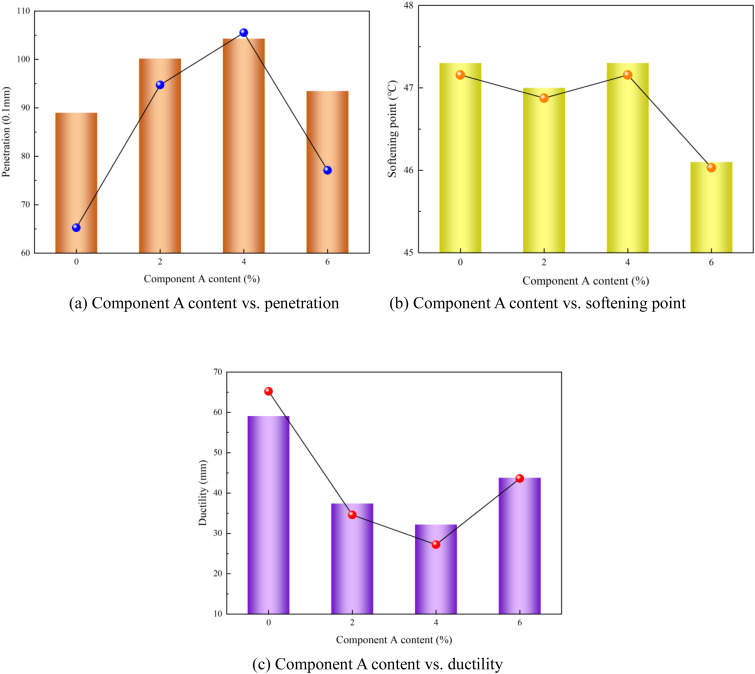
Component A content vs. modified asphalt performances (a) Component A content vs. penetration (b) Component A content vs. softening point (c) Component A content vs. ductility.

As shown in Fig 8, the component A content in the modified asphalt exerted a significant influence on penetration and ductility, whereas its effect on the softening point was relatively minor. When analyzing the impact of the component A content on the modified asphalt performance, the focus should be on its correlation with penetration and ductility. The relationship between component A content and modified asphalt performance is illustrated in Fig 10.

As shown in Fig 10, as the component A content increased, the penetration performance of the polyurethane/phosphogypsum-modified asphalt followed a V-shaped curve, initially increasing and then decreasing, and ductility exhibited the opposite trend of first decreasing and then increasing. This indicates that the viscosity of the modified asphalt was relatively optimized when the component A content was within the range of 2% to 4%. Based on comprehensive considerations, 2% component A content in the modified asphalt is recommended.

### 4.2. Effect of component B content on performance of modified asphalt

**Fig 11:**
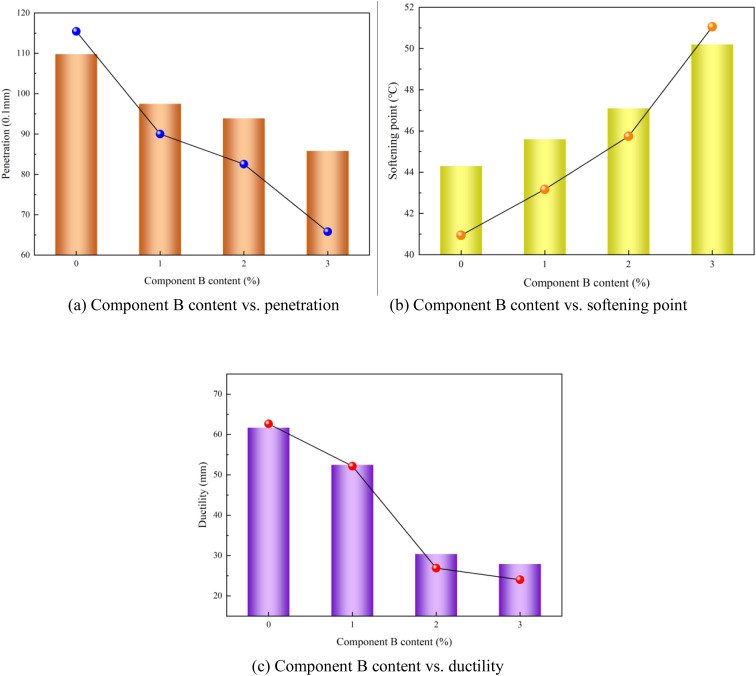
Component B content vs. modified asphalt performances (a) Component B content vs. penetration (b) Component B content vs. softening point (c) Component B content vs. ductility.

The component B content exerted the most significant influence on the performance of the polyurethane/phosphogypsum-modified asphalt. Notably, the extremum differences (range of variation) among the performance indicators (penetration, ductility, and softening point) caused by varying component B content were minimal, with numerical deviations not exceeding 0.1. Therefore, a holistic evaluation is required when analyzing the impact of component B content on the modified asphalt performance. The relationship between component B content and modified asphalt performance is illustrated in Fig 11.

As shown in Fig 11, increasing the component B content led to a gradual decline in the penetration performance and ductility of the polyurethane/phosphogypsum-modified asphalt and a progressive increase in the softening point. Based on a balanced evaluation of these opposing trends, a 1% component B content in the modified asphalt is recommended, as the overall performance of the modified asphalt with 1% component B content is preferable.

### 4.3. Effect of phosphogypsum content on performance of modified asphalt

**Fig 12:**
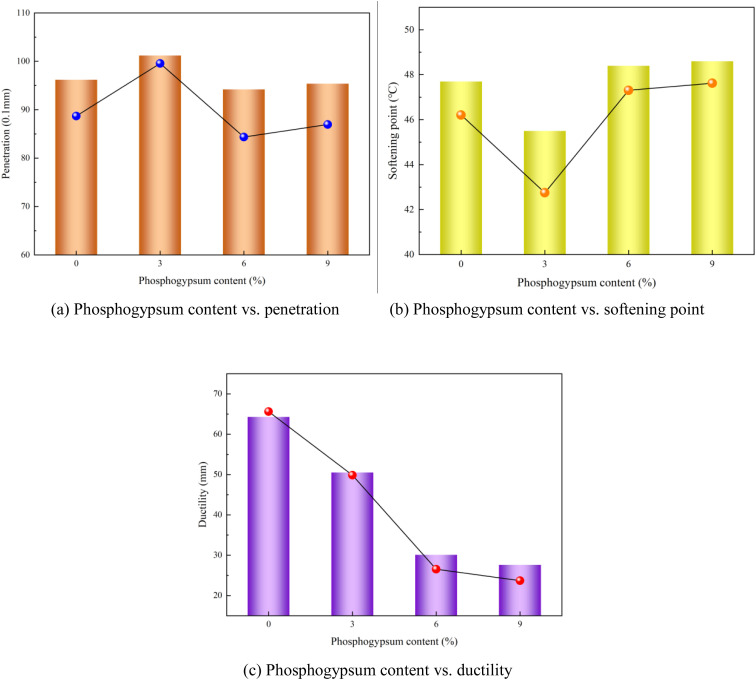
Phosphogypsum content vs. modified asphalt performances(a) Phosphogypsum content vs. penetration (b) Phosphogypsum content vs. softening point (c) Phosphogypsum content vs. ductility.

The phosphogypsum content exhibited a significant influence on the ductility of the modified asphalt; thus, this analysis primarily focuses on its correlation with the ductility of the modified asphalt. The relationship between the phosphogypsum content and modified asphalt performance is shown in Fig 12.

As indicated in Fig 12, increasing the phosphogypsum content led to a gradual decrease in the ductility of the polyurethane/phosphogypsum-modified asphalt. Notably, this declining trend slowed when the phosphogypsum dose exceeded 6%. Therefore, the recommended content in the modified asphalt should not exceed 6%.

### 4.4. Effect of coupling agent content on performance of modified asphalt

**Fig 13:**
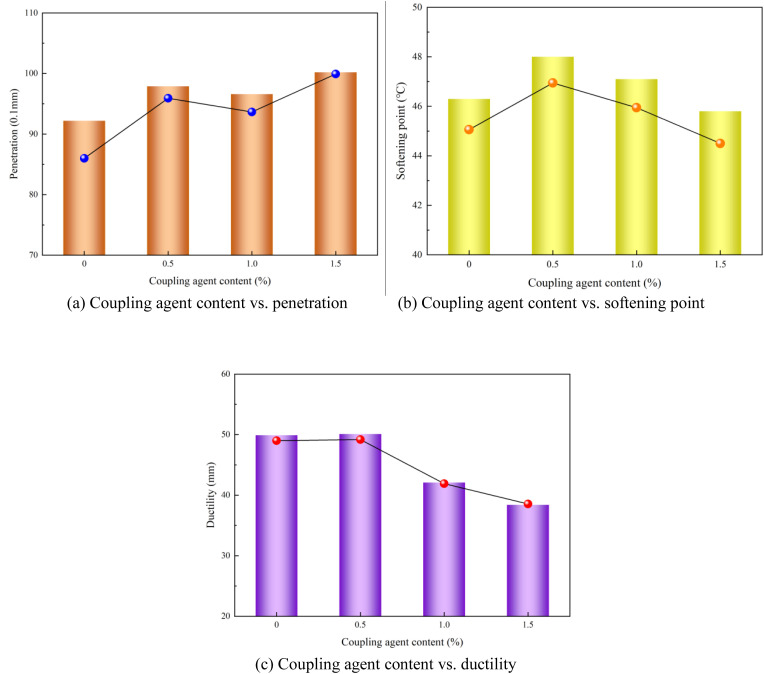
Coupling agent content vs. modified asphalt performances (a) Coupling agent content vs. penetration (b) Coupling agent content vs. softening point (c) Coupling agent content vs. ductility.

The extremum difference values (range of variation) in the modified asphalt performance caused by the coupling agent content ranged from 0.3 to 0.6, with ductility exhibiting the largest variation. The relationship between the coupling agent content and modified asphalt performance is shown in Fig 13.

As indicated in Fig 13, increasing the coupling agent content resulted in a progressive increase in the penetration performance, gradual decline in the ductility, and nonmonotonic trend (initially increasing and subsequently decreasing) in the softening point of the polyurethane/phosphogypsum-modified asphalt. Consequently, the recommended optimal coupling agent content in modified asphalt is 0.5%.

In summary, the polyurethane/phosphogypsum-modified asphalt demonstrated superior performance when formulated with 3% component A, 1% component B, 5% phosphogypsum, and 0.5% coupling agent. This composition is recommended as the optimal formulation for polyurethane/phosphogypsum-modified asphalt, balancing the penetration, ductility, and softening point to achieve enhanced mechanical and thermal properties.

## 5. Performance of polyurethane/phosphogypsum-modified asphalt and its mixtures

The optimal preparation process parameters and composition of the modified asphalt were used in this study to verify the performances of the polyurethane/phosphogypsum-modified asphalt and its mixtures.

### 5.1. Aging performance of composite-modified asphalt

The performances of the base asphalt and polyurethane/phosphogypsum composite-modified asphalt with the optimal composition were evaluated before aging, after short-term aging (85-min RTFOT), and after long-term aging (5-h RTFOT). The test results are presented in Table 7.

**Table 7:** Performance test results of different types of asphalt before and after aging.

Test items	Base asphalt					Modified asphalt				
	Normal	Short-term aging		Long-term aging		Normal	Short-term aging		Long-term aging	
	Test values	Test values	Change rate	Test values	Change rate	Test values	Test values	Change rate	Test values	Change rate
Penetration (0.1 mm)	90.2	49.6	45.0	31.8	64.7	88.9	53.3	40.0	37.6	57.7
Ductility (cm)	73.2	53.2	27.3	28.0	61.7	23.0	18.0	21.7	14.0	39.1
Softening point (°C)	45.8	51.9	13.3	62.6	36.7	47.5	52.2	9.9	61.6	29.7
Mean value (%)	--	--	28.5	--	54.4	--	--	23.9	--	42.2

As shown in Table 7, the polyurethane/phosphogypsum-modified asphalt exhibits decreased penetration and ductility but increased softening point compared to the base asphalt. The formation of polyurethane crosslinked networks significantly enhances the rigidity of the asphalt system, where the three-dimensional network structure restricts the free movement of asphalt molecules. And, the phosphogypsum act as rigid fillers uniformly dispersed in the asphalt matrix, further improving the overall stiffness through physical filling effects. And the average change rate of the modified asphalt properties after short-term aging was 23.9%, which was lower than that of the base asphalt (28.5%). Following long-term aging, the modified asphalt exhibited an average property change rate of 42.2%, which was significantly lower than the change rate of 54.4% observed in the base asphalt. This indicates that the composite modification using phosphogypsum and polyurethane effectively enhances the aging resistance of the asphalt, demonstrating a remarkable improvement in the long-term anti-aging performance [28–29].

### 5.2. Aging performance of modified asphalt mixtures

To further validate the aging resistance of the modified asphalt mixtures, specimens were subjected to short-term aging (4-h heating in a 135°C ventilated oven) and long-term aging (5-day heating in a 85°C ventilated oven). Subsequently, their high-temperature performance (Marshall stability and flow value) was evaluated and compared with that of unaged specimens. The test results are presented in Table 8.

**Table 8:** Performance test results of different types of asphalt mixtures before and after aging.

Test items	Base asphalt mixture					Modified asphalt mixture				
Normal	Short-term aging		Long-term aging		Normal	Short-term aging		Long-term aging	
Test values	Test values	Change rate	Test values	Change rate	Test values	Test values	Change rate	Test values	Change rate
Marshall stability (kN)	13.0	10.8	16.9	10.7	17.7	16.4	15.4	6.3	14.1	14.0
Flow value (0.1 mm)	3.32	2.87	13.6	2.35	29.2	3.05	2.71	11.1	2.37	22.3
Mean value (%)	--	--	15.3	--	23.5	--	--	8.7	--	18.2

Table 8 reveals that the modified asphalt mixture exhibited an average change rate of 8.7% in high-temperature performance after short-term aging, which was significantly lower than the average change rate of 15.3% observed in the base asphalt mixture. Following long-term aging, the modified mixture maintained superior performance, with an average change rate of 18.2%, compared with 23.5% for the conventional mixture. These results conclusively demonstrate the enhanced aging resistance of the modified asphalt mixture system.

## 6. Conclusions

The preparation process parameters and composition of polyurethane/phosphogypsum-modified asphalt were studied and the aging resistance of the modified asphalt and its mixtures was investigated. The key findings are summarized as follows:

(1) The influence of the preparation process parameters (such as the preparation temperature, shear rate, reaction time, and storage time) on the performance indicators (penetration, softening point, and ductility) was investigated based on orthogonal experimental analysis and the optimal parameters for the composite-modified asphalt were recommended. The results showed that the order of the influence of the different preparation process parameters on the performance of the modified asphalt is as follows: preparation temperature > storage time > reaction time > shear rate. The optimal preparation process parameters (130°C preparation temperature, 2000 rpm shear rate, 1.5 h reaction time, and 1.0 h storage time) of the polyurethane/phosphogypsum composite-modified asphalt were recommended based on the experimental results.
(2) The influence of different compositions on the performance parameters (such as the penetration, softening point, and ductility) of the polyurethane/phosphogypsum composite-modified asphalt was investigated and the optimal composition was recommended. The results confirmed that the order of influence of the composition on the modified asphalt performance is as follows: component B > component A > phosphogypsum > coupling agent. The polyurethane/phosphogypsum-modified asphalt with 3% component A, 1% component B, 5% phosphogypsum, and 0.5% coupling agent demonstrated superior performance.
(3) The performances of the polyurethane/phosphogypsum-modified asphalt and its mixtures were evaluated. The results showed that the average change rates of the modified asphalt properties after short- and long-term aging were 23.9% and 42.2%, respectively, which were lower than those of the base asphalt. Additionally, the modified asphalt mixture exhibited average change rates of 8.7% and 18.2% in high-temperature performance after short- and long-term aging, respectively, which were significantly lower than the average change rates of 15.3% and 23.5% observed in the base asphalt mixture.

## Supporting information

S1 File Test data.
(DOC)
