# Supplementary material for: Preparation process and properties of polyurethane/phosphogypsum-modified asphalt and its mixtures
Source: PLoS One. 2025 Jul 11;20(7):e0327312. doi: 10.1371/journal.pone.0327312 (PMC12250168; doi:10.1371/journal.pone.0327312)
Supplement: S1 File — Test data. (DOC) [file pone.0327312.s001.doc]

**This file includes all the test data.**

**Table 1 Technical indicators of 90#** base asphalt

| Test items | Test values | Required values |
| --- | --- | --- |
| 25°C Penetration (0.1mm) | 92.2 | 90~ 110 |
| 10°C Ductility (cm) | 112 | ≥25 |
| Softening point (℃) | 46.5 | ≥42 |

**Table 2 Technical indicators** of coarse aggregate

| Test items | Test values of different aggregates | | | Standard values |
| --- | --- | --- | --- | --- |
|  | 9.5-16mm | 4.75 - 9.5 mm | 2.36 - 4.75 mm |  |
| Density (g/cm3) | 2.672 | 2.699 | 2.700 | ≥2.6 |
| Water absorption (%) | 0.766 | 1.041 | - | ≤2.0 |

**Table 3 Preparation process parameter levels of** orthogonal experimental design

| Levels | Preparation temperature (°C) | Shear rate (rmp) | Reaction time (h) | Storage time (h) |
| --- | --- | --- | --- | --- |
| I | 120 | 1500 | 0.5 | 0.5 |
| II | 130 | 2000 | 1.0 | 1.0 |
| III | 140 | 2500 | 1.5 | 1.5 |
| IV | 150 | 3000 | 2.0 | 2.0 |

**Table 4 The experimental schemes of different preparation process parameters**

| No. | Preparation temperature (°C) | Shear rate (rmp) | Reaction time (h) | Storage time (h) |
| --- | --- | --- | --- | --- |
| 1 | 120 | 1500 | 0.5 | 0.5 |
| 2 | 120 | 2000 | 1.0 | 1.0 |
| 3 | 120 | 2500 | 1.5 | 1.5 |
| 4 | 120 | 3000 | 2.0 | 2.0 |
| 5 | 130 | 2000 | 0.5 | 1.0 |
| 6 | 130 | 2500 | 1.0 | 0.5 |
| 7 | 130 | 1500 | 1.5 | 2.0 |
| 8 | 130 | 3000 | 2 | 1.5 |
| 9 | 140 | 3000 | 0.5 | 1.5 |
| 10 | 140 | 2500 | 1.0 | 2.0 |
| 11 | 140 | 2000 | 1.5 | 0.5 |
| 12 | 140 | 1500 | 2.0 | 1.0 |
| 13 | 150 | 2000 | 0.5 | 2.0 |
| 14 | 150 | 1500 | 1.0 | 1.5 |
| 15 | 150 | 3000 | 1.5 | 1.0 |
| 16 | 150 | 2500 | 2.0 | 0.5 |

**Table 5 C**omposition levels of orthogonal experimental design

| Levels | Component A content (%) | Component B content B (%) | Phosphogypsum content (%) | Coupling agent content (%) |
| --- | --- | --- | --- | --- |
| I | 0 | 0 | 0 | 0 |
| II | 2 | 1 | 3 | 0.5 |
| III | 4 | 2 | 6 | 1.0 |
| IV | 6 | 3 | 9 | 1.5 |

**Table 6 The experimental schemes of different compositions of modified asphalt**

| No. | Component A content (%) | Component B content B (%) | Phosphogypsum content (%) | Coupling agent content (%) |
| --- | --- | --- | --- | --- |
| 1 | 0 | 0 | 0 | 0 |
| 2 | 0 | 1 | 3 | 0.5 |
| 3 | 0 | 2 | 6 | 1.0 |
| 4 | 0 | 3 | 9 | 1.5 |
| 5 | 2 | 2 | 0 | 1.5 |
| 6 | 2 | 3 | 3 | 0 |
| 7 | 2 | 0 | 6 | 1.5 |
| 8 | 2 | 1 | 9 | 1.0 |
| 9 | 4 | 3 | 0 | 1.0 |
| 10 | 4 | 2 | 3 | 1.5 |
| 11 | 4 | 1 | 6 | 0 |
| 12 | 4 | 0 | 9 | 0.5 |
| 13 | 6 | 1 | 0 | 1.5 |
| 14 | 6 | 0 | 3 | 1.0 |
| 15 | 6 | 3 | 6 | 0.5 |
| 16 | 6 | 2 | 9 | 0 |

**Table 7** Performance test results of different types of asphalt before and after aging

| Test items | Base asphalt | | | | | Modified asphalt | | | | |
| --- | --- | --- | --- | --- | --- | --- | --- | --- | --- | --- |
|  | Normal | Short-term aging | | Long-term aging | | Normal | Short-term aging | | Long-term aging | |
|  | Test values | Test values | Change rate | Test values | Change rate | Test values | Test values | Change rate | Test values | Change rate |
| Penetration (0.1mm) | 90.2 | 49.6 | 45.0 | 31.8 | 64.7 | 88.9 | 53.3 | 40.0 | 37.6 | 57.7 |
| Ductility (cm) | 73.2 | 53.2 | 27.3 | 28.0 | 61.7 | 23.0 | 18.0 | 21.7 | 14.0 | 39.1 |
| Softening point (℃) | 45.8 | 51.9 | 13.3 | 62.6 | 36.7 | 47.5 | 52.2 | 9.9 | 61.6 | 29.7 |
| Mean value (%) | -- | -- | 28.5 | -- | 54.4 | -- | -- | 23.9 | -- | 42.2 |

**Table 8 Performance test results of different types of asphalt mixtures** before and after aging

| Test items | Base asphalt mixture | | | | | Modified asphalt mixture | | | | |
| --- | --- | --- | --- | --- | --- | --- | --- | --- | --- | --- |
|  | Normal | Short-term aging | | Long-term aging | | Normal | Short-term aging | | Long-term aging | |
|  | Test values | Test values | Change rate | Test values | Change rate | Test values | Test values | Change rate | Test values | Change rate |
| Marshall stability (kN) | 13.0 | 10.8 | 16.9 | 10.7 | 17.7 | 16.4 | 15.4 | 6.3 | 14.1 | 14.0 |
| Flow value (0.1mm) | 3.32 | 2.87 | 13.6 | 2.35 | 29.2 | 3.05 | 2.71 | 11.1 | 2.37 | 22.3 |
| Mean value (%) | -- | -- | 15.3 | -- | 23.5 | -- | -- | 8.7 | -- | 18.2 |
